# Is Working from Home a Blessing or a Burden? Home Demands as a Mediator of the Relationship Between Work Engagement and Work-Life Balance

**DOI:** 10.1007/s11482-022-10084-6

**Published:** 2022-07-22

**Authors:** Fabian Onyekachi Ugwu, Ibeawuchi K. Enwereuzor, Jens Mazei

**Affiliations:** 1 Department of Psychology, Alex Ekwueme Federal University Ndufu-Alike, Abakaliki, Ebonyi State Nigeria; 2 Institute for Psychology, TU Dortmund University, Dortmund, Germany; 3 Department of Psychology, University of Nigeria, Nsukka, Nigeria

**Keywords:** Working from home, Work engagement, Home demands, Work-life balance, Nigeria

## Abstract

As COVID-19 pandemic made its incursion into the world of work in early 2020, many employees were compelled to work from home to slow down the transmission of the disease. Since then, it has been asked whether working from home is a blessing or a burden. We respond to this question by building on the Affective Events Theory to examine whether work engagement is related to work-life balance (WLB), and whether home demands mediate this relationship, using data from 219 knowledge workers drawn from universities in the South-eastern region of Nigeria primarily working from home when they were surveyed. Results of regression analysis using PROCESS macro showed that work engagement related positively to home demands; in turn, home demands related negatively to WLB. The results further revealed that work engagement related negatively to WLB and that home demands mediated the negative work engagement-WLB connection. Theoretical as well as practical implications of the study are discussed, limitations are highlighted, and suggestions for future research are outlined.

## Introduction

Over the past two decades, the boundaries between individuals’ work and nonwork life have become narrow (Kinnunen et al., 2014). This condition has been aggravated since COVID-19 made its incursion into the world of work. Vyas and Butakhieo (2021) reported that COVID-19 is a new virus that belongs to the coronavirus family that shares similar symptoms with severe acute respiratory syndrome (SARS) that swept across Asia in 2002 and the Middle East ten years later. The virus and its implications for people’s daily life appear to have shocked the world. Despite various measures that have been adopted to reduce the spread of the virus, such as the use of nose-to-mouth masks, physical distancing, and regular hand sanitizing (Centre for Health Protection [CHP], 2020), the virus continues to spread quickly. Since the outbreak of the virus, more than 60 million people are reported to have been infected, and over 1.4 million deaths have occurred until November 26, 2020, with the number of casualties continuing to soar, causing the outbreak to be declared a global health pandemic on January 31, 2020 (World Health Organization, 2020). The threat to life posed by the virus forced governments all over the world to impose strict lockdowns that brought about closure of non-essential services. The Nigerian government also imposed and enforced restrictions that prohibited public gathering. Hiekel and Kühn (2021) reported that these restrictions affected *employees* more than they did to any other group, forcing them to work from home. Working from home (WFH), which is a relatively old concept that dates to 1973, also referred to as “telecommuting” or “telework” (Messenger & Gschwind, 2016), has resurfaced, and is commonly labeled as “the new normal” in many organizations (Abdel Hadi et al., 2021). In Nigeria, where the current study was conducted, however, WFH was relatively unknown until these restrictions were imposed. Being compelled to work from home to keep organizations running may have various and novel consequences on the private lives of employees. Hence, Nigeria offers an exciting context in which the unique effects of *newly introduced* WFH can be examined.

WFH can make meaningful difference for employees, especially when considering past changes in family structures that opened the door for greater participation of women in the world of work (Peeters et al., 2005), which provide a sense of shared responsibility for family duties among couples. Given such shared responsibilities, conducting work from home may impact negatively on people’s work-life balance (WLB). This is because WFH can create increased porosity on the boundaries between work and nonwork domains (Grant et al., 2013), which makes it challenging for individuals to simultaneously manage work, home, and personal lives (Jones et al., 2006). Hence, “weak” boundaries between employees’ work and nonwork activities may lead to conflicts (Bakker et al., 2008). Thus, in our research, we illuminate the important challenges encountered by employees due to COVID-19—working from home—by examining how work engagement is related to home demands and WLB. Moreover, we elaborate on the broader implications of these changes to people’s “patterns of daily life” for societies and its organizations (Layton & Domegan, 2021, p. 4).

One occupational group that may be particularly vulnerable to adverse work-related consequences is academics (Listau et al., 2017). Although academics appear to be committed and satisfied with their work (Harman, 2003) and are also intrinsically motivated due to their job autonomy and flexibility (Bellamy et al., 2003), evidence suggests that the workload of academics is rather high (Harman, 2003; Listau et al., 2017). The job of academics is complex and multifaceted—including teaching, research, and community development. Academics also may have a tougher time than employees in other professions in terms of keeping their WLB because their work is immensely “open-ended” (Wortman et al., 1991) and consists of a variety of roles with partly opposing demands (Fisher, 1994). Superfluous administrative work often has been added to their list of duties, making their job even more complicated. In addition, their tasks typically have timeline (Ingusci et al., 2021).

Altogether, academics must work hard and fast to accomplish their numerous and complex tasks within a restricted time span (Bakker & Demerouti, 2017; Ingusci et al., 2021), hence, to accomplish their work, academics often work long hours and overtime (Houston et al., 2006). Working for extended periods to get work done may be more pronounced and impactful when employees are compelled to work from home, which could deplete their resources and lead to negative work-related outcomes (Listau et al., 2017; Van Tonder & Fourie, 2015), including work-home conflict (Bell et al., 2012) and potentially other negative work-related outcomes (Hakanen et al., 2008). Exploring the impact of work engagement on the WLB of academics, therefore, appears to be particularly important. Achieving a high satisfaction at home and workplace (Campbell Clark, 2000) in which time allocated to work and nonwork are roughly equal (Kirchmeyer, 2000), may determine how successful employees would be in achieving WLB. WLB refers to individual’s capability to realize the goals and/or deal with job demands to enhance personal life and attain satisfaction in all aspects of life (Bulger & Fisher, 2012). WLB has been more elaborately captured as employees’ perceptions of how efficiently they manage work and nonwork roles in relation to the value they place on their private lives, objectives, and desires (Casper et al., 2018; Greenhaus & Allen, 2011; Haar, 2013; Haar et al., 2019; Valcour, 2007).

WFH can have effects on two broad domains – outcomes in the work and life domains (Vyas & Butakhieo, 2021). It is demonstrated that WFH has positive impacts on work domain such as performance and work engagement (Gerards et al., 2018; Grant et al., 2019; Purwanto et al., 2020; ten Brummelhuis et al., 2012). Since WFH gives rise to work engagement, and work engagement is related positively to work-family conflict (Halbesleben et al., 2009) and generally to work-life conflicts (Borst et al., 2020), due to its resource-depleting capabilities, we reasoned that the “dark side” of work engagement could be responsible for people’s difficulty in reconciling work and personal life. Moreover, although Wood et al. (2020) reviewed 12 empirical studies on the link between work engagement and WLB factors, including work–family imbalance, work–to–family and family–to–work conflicts, and work–family spillover (e.g., Ilies et al., 2017; Vîrgă et al., 2015), surprisingly, none of these studies considered *home demands* as a pathway through which work engagement is related to WLB. Therefore, the question that has not been fully answered is, *why* does employee work engagement relate to WLB? Considering that home demands is particularly important in the present study because employees who are compelled to work from home will likely be entangled with family duties while performing their job. The current study hence explores whether work engagement is related to WLB via home demands.

Furthermore, we study this question in Nigeria, a context that has been rarely considered. Despite mounting research on WLB conducted in Western-European contexts, further development of the construct is required (Greenhaus & Allen, 2011; Haar et al., 2019; Schnettler et al., 2021). Shockley et al. (2017) estimated that only 10% of the studies on work–family research that focused on WLB gave attention to contexts. Therefore, investigating WLB in other cultures is important because individuals’ cultural contexts have varying impacts on WLB (Greenhaus & Powell, 2017; Haar et al., 2019; Ollier-Malaterre & Foucreault, 2017). Studying WLB only in Western-European cultures could lead to a narrow or simplified understanding of WLB (e.g., Haar et al., 2019; Shockley et al., 2017). In fact, there is meaningful cultural variation across Western-European contexts, where much of the previous studies were conducted and African cultures, in terms of individualism versus collectivism orientations. Particularly, collectivistic African culture is characterized by the integration of individuals into strong, cohesive in-groups, with individuals receiving support from, and making contributions to supporting others. Conversely, Western-European culture is typically more individualistic, such that individuals belong to a loosely knit society where more value is placed on the self (Hofstede, 1997). Hence, it becomes imperative to pay close attention to cultural differences to gain deeper understanding of the link between job demands related to WFH and the presence of WLB.

### Theoretical Background and Development of Hypotheses

High job demands can exhaust individuals’ resources and make them vulnerable to negative job outcomes (Bakker & Demerouti, 2007; Hakanen & Roodt, 2010). Hence, we argue that work engagement, a potential job demand (see below), can deplete role resources and exert negative impact on WLB. Also, home demands (e.g., taking on family roles and responsibilities) may further deplete the already depleted resources and serve as a pathway through which work engagement indirectly affects WLB (Chernyak-Hai & Tziner, 2016; Ilies et al., 2017; Mache et al., 2016; Wood et al., 2020). Given that commitment to work and family demands require enormous energy and emotional investments, they deplete role resources (Rothbard, 2001). When resources are lacking, there will be fewer left for individuals to cope with personal life.

**Fig. 1:**
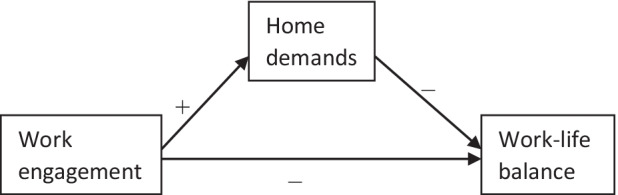
Conceptual model

Our study is based on the Affective Events Theory (AET; Weiss & Cropanzano, 1996). At the heart of AET is the assertion that one’s affective work experiences and events directly impact on behaviors and attitudes, suggesting that a loss of resources occasioned by work engagement (see below), through home demands, may leave employees exhausted and with insufficient resources, which leads to affective negative response regarding WLB (Weiss & Cropanzano, 1996). This could be the reason why Ashkanasy et al. (2002) stated that “AET is unique in explicating what happens inside the ‘black box’ between the work environment and subsequent employee attitudes and behavior” (p. 323). More specifically, we speculate that work engagement by academics may have a harmful effect on their WLB via home demands, principally due to resource loss (Fig. 1).

### Work Engagement and Work-Life Balance

Work engagement, defined as a state of mind with vigor, dedication, and absorption as its primary features (Bakker & Albrecht, 2018; Schaufeli et al., 2002), has arguably attracted the most research attention in occupational and management literature over the last two decades. The popularity of work engagement is down to its positive effects on a variety of work outcomes, both on the individual and organizational levels (Christian et al., 2011; Nutcache, 2019). The strength of work engagement in predicting desirable organizational outcomes, including job satisfaction, organizational citizenship behavior (OCB), work commitment, turnover intention, and innovative work behavior has been demonstrated in several meta-analyses (e.g., Borst et al., 2020; Christian et al., 2011; Harter et al., 2002; Neuber et al., 2021; Sari et al., 2020). However, contrary to the assumption of Christian et al. (2011) and Nutcache (2019) that work engagement consistently leads to positive outcomes both for the employees and the organization, there has been a recent surprising twist in the outcomes of work engagement research, with studies uncovering also negative individual outcomes (e.g., Baethge et al., 2021; Halbesleben et al., 2009; Junker et al., 2021). Recent empirical evidence (e.g., Baethge et al., 2021; Junker et al., 2021) has challenged the assumption of universal positive impacts of work engagement by revealing that work engagement is also related to several *negative* consequences that tend to threaten the organization and its members. For instance, work engagement is found to relate to exhaustion (e.g., Cole et al., 2012; Mäkikangas et al., 2017; Moeller et al., 2018). Work engagement is equally found to have a U-shaped, or curvilinear relationship with psychological distress (Shimazu et al., 2018). Work engagement can also lead to greater turnover intentions (Caesens et al., 2016), more work-family conflict (Halbesleben et al., 2009). Work engagement can also negatively influence individual’s private life (e.g., Halbesleben, 2010; Timms et al., 2015).

Various reasons have been advanced to explain the negative consequences of work engagement on job and individual outcomes. These reasons are linked to the three components of work engagement – vigor, dedication, and absorption. For example, vigor entails employees’ readiness to invest energy and commitment into their job and be resolute in the face of difficult challenges (Timms et al., 2015; Tims et al., 2012). As a result, employees devote reasonable amounts of resources to work (Schaufeli et al., 2006), and in the process they exhaust the resources required to deal with family roles, resulting in difficulties to balance work and private life (Borst et al., 2020). The component of dedication entails employees’ high psychological involvement in their work, where they have high sense of enthusiasm (Schaufeli et al., 2002), making engaged employees to overlook signs of fatigue (Sonnentag et al., 2010) and ignore sickness to continue working (Miraglia & Johns, 2016). Finally, the absorption dimension of work engagement, which refers to a condition where employees are fully immersed in work and where they find it challenging to disconnect from work (Mauno et al., 2007) could lead them to skip breaks at work (Bakker & Oerlemans, 2016), which may again lead to difficulties in attaining WLB. Flow at work – a construct that has resemblance with absorption has also been found to be related to exhaustion (Demerouti et al., 2012; González-Roma et al., 2006).

Work engagement has also been linked to workaholism – an inner drive to work compulsively and more than what is generally expected (Clark et al., 2014). Such behaviors limit time and energy needed to perform nonwork activities, which increases exhaustion over time (Junker et al., 2021). Another, related explanation for potential negative effects of work engagement on WLB can be found in the resource scarcity hypothesis. The amounts of resources that individuals possess are limited (e.g., time and energy) and as such, handling numerous roles (e.g., work and home responsibilities), while trying to maintain WLB, is challenging as each depends on the same scarce resources (Shimazu et al., 2010). Like the impact of job demands, work engagement, which involves sustained sympathetic arousal or activation (Baethge et al., 2021; Nes et al., 2005; Shimazu et al., 2018) entails resource investment, and, in the process, it can physiologically deplete individuals’ resource base (Baethge et al., 2021; Sonnentag, 2001), leaving employees with insufficient resources to harmonize work and personal life.

Although studies are beginning to focus on the “dark side” of work engagement, critical questions remain unanswered. For instance, whether and how the “dark side” of work engagement emerges, such that it negatively affects individuals’ WLB, has not been thoroughly investigated. Therefore, our goal is to extend our understanding of the “dark side” of work engagement regarding work-family conflict by examining a specific pathway through which it is negatively related to WLB. A focus on WLB in this study was borne out of concern that current pervasive job demands in the work and home contexts tend to threaten WLB, which is known to play dominant role in general satisfaction (Greenhaus et al., 2003; Keyes, 2002). It has also been documented that WLB exert influence on individuals’ work life by enhancing job satisfaction and commitment and, at the same time, by lowering stress (e.g., Ford et al., 2007; Kossek et al., 2014; Liu et al., 2021). To further buttress the importance of WLB, it has been considered as moderating and mediating variables in many studies (e.g., Chiang et al., 2010; Rashmi & Kataria, 2021; Santhanam et al., 2020). However, employees’ experience of WLB is threatened by insufficient resources to cope with activities in the nonwork domain. Hämmig and Bauer (2009) asserted that a threat to WLB is a risk factor affecting mental health, job performance, and the family of employees (Anwar & Shahjad, 2011; Jayanthi & Vanniarajan, 2012). Impediments on WLB have also been implicated in general stress and burnout (Hämmig et al., 2012), withdrawal behavior, and unnecessary sick leave (Hughes & Bozionelos, 2007). These findings underscore the importance of expending efforts to enhance WLB among employees. Drawing on this evidence, we propose that:

- *Hypothesis 1:* Work engagement is positively related to home demands.
- *Hypothesis 2:* Home demands relates negatively to WLB.
- *Hypothesis 3:* Work engagement relates negatively to WLB.

### Mediating Role of Home Demands

Abdel Hadi et al. (2021) stated that “WFH might be a blessing and a burden at the same time because it may offer increased flexibility to deal with multiple (conflicting) demands in the work-home interface” (p. 532). Home demands might equally become important in accounting for negative employee behavior, as these employees invest more resources – time on WFH (Abdel Hadi et al., 2021; Konradt et al., 2003). Home demands refer to aspects of domestic life that include enduring cognitive and emotional commitment or ability and are therefore linked to some psycho physiological outcomes (Nel et al., 2012). Home demands have also been referred to as employees’ general perceptions of the amount and strength of family roles (Boyar et al., 2007). Home demands entail caregiving to every member of the family, including the elderly (Yang et al., 2000). These roles have been associated with increased work-family conflict and reduced work-family enrichment (Voydanof, 2005).

Indeed, under conditions of WFH, employees are expected to harmonize demands from job and home (McNaughton et al., 2014). However, most previous studies tend to have subsumed and discussed home demands under job demands, but researchers have stated that, although job and home domains affect each other (Geurts & Sonnentag, 2006), they are conceptually different (Peeters et al., 2005; Sonnentag & Zijlstra, 2006). As a result, scholars have argued that not until home demands receive significant research attention as job demands, would we have a more comprehensive understanding of these demands constructs (Mostert, 2009; Van Aarde & Mostert, 2008). High home demands (e.g., housekeeping and childcare; Choi, 2008) lead employees to devote more resources to family, leaving these employees with fewer resources to allocate to their private lives, which may limit the opportunity to achieve WLB. Although home demands have been reported to create good mood in employees, especially when these employees have high level of motivation towards home demands (Pennonen, 2011), they have also been reported to be related to excessive constraints in addition to work (Peeters et al., 2005). Studies (e.g., Mostert, 2009; Van Aarde & Mostert, 2008) found home demands to be related to home interference and poor health. Peeters et al. (2005) found that home and job demands are related to burnout. Shimazu et al. (2010) found that home demands were partly directly and indirectly related to distress via family work conflicts. Although most employees experience home demands, which may have become more audacious under conditions of WFH, yet studies that focused on the outcomes of this construct are lacking. Moreover, as far as we know, studies that explored home demands as a pathway in work engagement-WLB link are nonexistent. Based on the above arguments, we propose that:

- *Hypothesis 4:* Home demands mediate the negative relationship between work engagement and WLB.

## Method

### Participants and Procedure

Participants for the study were of 219 teaching staff members from various universities in Southeast, Nigeria. All the data were collected between the months of June and November 2020. During this period, the federal government of Nigeria imposed restrictions to public places and the schools including universities were mostly affected. Universities were shut down and academics were required to work remotely from home. The lockdown also affected leisure routines as physical distancing was emphasized. Therefore, the respondents were recruited via social media (different WhatsApp group platforms) exclusively across universities within the Southeast, Nigeria. The age of participants ranged from 31 to 56 years with a mean age of 46.55 years (*SD* = 5.66). Males (*n* = 62 (28%) and females (*n* = 157 (72%) participated in the study. After individuals responded positively to the informed consent about their readiness to take part in the study, they had access to the set of the questionnaires that included items on work engagement, home demands, and WLB. The participants’ demographic profile is represented in Table 1.

**Table 1:** Participants’ Demographic Profile

Demographics	Frequency	Percentage
Gender		
Male	62	28%
Female	157	72%
Marital status		
Single	34	15.5
Married	185	84.5
No. of children		
0–3	125	57.1%
4–6	94	42.9%
Age		
30–39	27	12.3%
40–49	121	55.3%
50–59	71	32.4%

*N* = 219

### Measures

All the scales for the study were adapted to the lockdown situation. Specifically, respondents were asked to respond to the scales while bearing in mind the challenges/experiences they face WFH due to the national lockdown engendered by COVID-19 pandemic. All the items used in assessing the constructs are presented in the Appendix.

### Antecedent Variables

We measured *work engagement* with the 9-item Utrecht Work Engagement Scale (Schaufeli & Bakker, 2003). Higher scores on the scale indicate a higher level of work engagement. Cronbach’s α of 0.79 was found for the current study.

*Home demands* was assessed with the 10-item scale (Peeters et al., 2005). Higher scores indicate a higher experience of home demands. Cronbach’s α of 0.83 was found for the current study.

### Outcome Variable

We measured WLB with the WLB Scale (Brough et al., 2014). Higher scores on the scale indicate higher WLB. Cronbach’s α of 0.87 of the scale was found for the present study.

### Strategy for Analyses

We used SPSS v25 to examine means and standard deviations (descriptive statistics) and correlations of the variables of interest to check whether any personal variable was significantly correlated with core study variables, so that these variables could be incorporated as a covariate during hypotheses testing. In addition, the hypothesized model was tested with ordinary least squares (OLS) regression using PROCESS macro (Hayes, 2018). Specifically, Model 4 of the PROCESS macro was used based on 5000 bootstrapped samples. Bootstrapping is a statistical procedure that entails resampling and building a sampling distribution from which confidence intervals (CIs) can be constructed, even if the sampling distribution is not normal (Hayes, 2018). The CIs (i.e., LLCI and ULCI) were used as the bases for determining the significance of the hypothesized relationships. To be significant, zero should not be within the ranges of the CIs.

## Results

### Descriptive Statistics and Intercorrelations

Table 2 reported the descriptive statistics and intercorrelations among the study variables. All the personal variables (i.e., gender, marital status, no. of children, and age) did not significantly correlate with WLB. They were, thus, dropped from successive analyses. All the key variables significantly correlated with WLB. Specifically, work engagement (*r* = −0.50, *p* < .001) and home demands (*r* = −0.48, *p* < .001) were negatively correlated with WLB.

**Table 2:** Means, Standard Deviations, and Correlations among the Study Variables

	Variable	*M*	*SD*	1	2	3	4	5	6
1	Gender	–	–	–					
2	Marital status	–	–	0.01	–				
3	No. of children	–	–	−0.01	0.69^***^	–			
4	Age	46.55	5.66	−0.11	0.54^***^	0.78^***^	–		
5	Work engagement	43.16	4.57	−0.03	0.04	−0.06	0.02	–	
6	Home demands	30.17	3.71	−0.11	0.01	−0.03	0.01	0.47^***^	–
7	Work-life balance	8.80	1.13	0.01	−0.04	−0.01	−0.03	−0.50^***^	−0.48^***^

*N =* 219. ^***^ = *p* < .001 (two-tailed). Gender was coded 0 = male, 1 = female; marital status: 1 = single, 2 = married; no. of children was coded based on actual number of children that each participant has such that higher scores represent a greater number of children; age was coded using number of years, such that higher scores represent older age. The remaining variables were coded such that higher scores represent higher values of the particular construct

### Hypothesis Testing

The OLS regression result presented in Table 3 shows that work engagement was positively related to home demands (*a* = 0.380, *p* < 0.001, 95% CI [0.284, 0.476]). Therefore, Hypothesis 1 was supported. Home demands were negatively related to WLB (*b* = −0.095, *p* < 0.001, 95% CI [−0.133, −0.057]). Therefore, Hypothesis 2 was also supported. Work engagement was negatively related to WLB (*c*’ = −0.087, *p* < 0.001, 95% CI [−0.118, −0.056]). Therefore, Hypothesis 3 was supported as well (Table 3).

**Table 3:** OLS Regression for Direct Relationship

	*M* (Home demands)						*Y* (Work-life balance)					
Antecedent		Coefficient	*SE*	*p*	95% CI			Coefficient	*SE*	*p*	95% CI	
					LLCI	ULCI					LLCI	ULCI
*X* (Work engagement)	*a*	0.380	0.049	< 0.001	0.284	0.476	*c*’	−0.087	0.016	< 0.001	−0.118	−0.056
*M* (Home demands)		–	–	–	–	–	*b*	−0.095	0.019	< 0.001	−0.133	−0.057
constant	*i*_*M*_	13.767	2.110	< 0.001	9.609	17.926	*i*_*Y*_	15.418	0.660	< 0.001	14.117	16.719
		*R*^2^ = 0.220 *F*(1, 217) = 61.139, *p* < 0.001						*R*^2^ = 0.321 *F*(2, 216) = 51.045, *p* < 0.001				

*LL* lower limit, *UL* upper limit, *CI* confidence interval, *Coeff.* coefficient, *SE* standard error. Results were based on 5000 percentile bootstrapped samples

The indirect relationship of work engagement on WLB through home demands was negative, and the CI did not include zero (*ab* = −0.036, 95% CI [−0.053, −0.021]). This indicates that home demands negatively mediated the relationship between work engagement and WLB. These results support Hypothesis 4 (Table 4).

**Table 4:** OLS Regression for Indirect Relationship

Pathway	Coefficient	BootSE	95% CI	
			BootLL	BootUL
Work engagement ➝ Home demands ➝ WLB	−0.036	0.008	−0.053	−0.021

*WLB* work-life balance. Results were based on 5000 percentile bootstrapped samples

## Discussion

The current study investigated work engagement-WLB connection, and the mediating role of home demands in this relationship among knowledge workers under conditions of WFH. Consistent with our predictions, the results revealed that work engagement was related positively to home demands. The results also showed that home demands related negatively to WLB, and that work engagement related negatively to WLB. The results further showed that home demands mediated the negative work engagement-WLB link. Altogether, all the hypothesized relationships in the study were supported. The found positive relationship between work engagement and home demands is expected because, while employees are made to work from home, chances are that work, as well as family roles distorted the borders between work and private life, which, in turn, permitted such roles to blend, thereby explaining the positive relationship found between work engagement and home demands.

Work engagement was found to be related negatively to WLB. This finding was expected because, in addition to the high activation that characterizes work engagement, engaged employees devote large amounts of resources to their jobs, which hence become depleted (Rothbard, 2001). In this sense, employees can be left with insufficient resources to balance work and personal life. Another possible explanation could be that university employees WFH work compulsively and under tight schedules as well as intense pressure to meet deadlines, and the additional home demands such as shared responsibilities with spouses could result in employees having less spare time for leisure activities that could have been necessary in managing their work and private lives. Our finding aligns with previous studies that linked work engagement to work-family conflict (Chen & Huang, 2016; Halbesleben et al., 2009; Rantanen et al., 2013), work-life conflicts (Borst et al., 2020), and burnout (Chernyak-Hai & Tziner, 2016). Yet, this finding conflicts with studies that found work engagement to be related to job satisfaction and low work-family conflict (Burke et al., 2013). The result is also in conflict with other studies (e.g., Culbertson et al., 2012) that positively related work engagement to family life and successful integration of work and family (Karatepe & Demir, 2014), to work–family enrichment (Chen & Powell, 2012; Qing & Zhou, 2017), to work-family facilitation (Bakker et al., 2014), and to work-family balance (Ilies et al., 2017). Altogether, it appears that work engagement has multiple effects (both positive and negative).

Furthermore, the present study provides support for the proposed mediating role of home demands in the negative relationship between work engagement and WLB. Specifically, work engagement had an indirect relationship with WLB via home demands. Employees invest significant resources to perform both work and family roles, which impose substantial demands on the employees. Therefore, work-related demands and home demands seem to deplete individuals’ resource base, which may overwhelm employees, leaving them with fewer resources to allocate to personal lives, which in turn makes it difficult for them to achieve WLB. These findings can be understood in light of the Affective Events Theory (Weiss & Cropanzano, 1996), which suggests that, due to loss of resources engendered by work engagement, and exacerbated by home demands, employees’ affective work experiences directly impact their behaviors and attitudes and, thus, leads to difficulties in achieving WLB. This finding is supported by prior studies that reported that home demands relate to excessive constraints in addition to work (e.g., Peeters et al., 2005), to higher work-family conflict, and lower work-family enrichment (Voydanof, 2005). The present finding also tends to be consistent with other studies (e.g., Mostert, 2009; Van Aarde & Mostert, 2008) that found home demands to be related to home-work interference and ill health (e.g., Shimazu et al., 2010), as well as burnout (e.g., Peeters et al., 2005). This finding also tends to agree with research (e.g., Abdel Hadi et al., 2021) suggesting that daily home demands during telework are positively related to emotional exhaustion and to perceived stress (Konradt et al., 2003).

### Theoretical Implications

Our study builds on existing WLB research by considering the Nigerian context where similar studies have not been thoroughly investigated (Amazue & Onyishi, 2016). An important contribution of our study rests on our finding that the negative link between work engagement and WLB is mediated by home demands. This finding shows that work engagement and home demands drain energy resources and due to insufficient resources, it becomes difficult to balance work and personal life. Additional theoretical contribution is that it is difficult to attain WLB under highly demanding conditions. In sum, this study extends our understanding of how work and home demands may become bottleneck for the achievement of WLB and highlights the need for calls for adequate workplace interventions or personal resources that may help to mitigate the relationships between demands and WLB (Schieman et al., 2009; Straub, 2012).

### Practical Implications

This study added to the accumulating evidence that employees who are work engaged experience negative side effects, in the form of difficulties in balancing work and personal lives. These difficulties were explained by the demands that employees experience at home. Therefore, in practical terms, our findings recommend a need for management to develop intervention techniques or programs that will stimulate work engagement without negatively affecting the personal lives of employees. Even when employees are WFH, such programs could be designed in a way that suggest or allows free time that would enable employees to engage in leisure activities that may enhance their WLB. In addition, work–family programs, such as on-site day care (Halbesleben et al., 2009), which have been captured in high-commitment work arrangements (Osterman, 1995), may be a viable strategy that may help to mitigate possible negative consequences of work engagement. Management can also encourage employees to strive for satisfactory balance between engagement, home demands, and personal life.

Furthermore, the role of adaptive coping skills, such as seeking instrumental support from the organization and emotional social support from a partner (Amazue & Onyishi, 2016; Carless & Whintle, 2007; Epie, 2010) can be vital as employees seek to cope with demands. Management can also encourage employees to adopt a problem-focused coping strategy (Pienaar, 2008), which entails having active attitudes and the knack to adapt to diverse roles of both work and life domains (Zheng et al., 2016). This coping strategy can be beneficial because individuals with a tendency for positive evaluations of difficult situations generally manage opposing job demands and personal life better (Greenhaus & Powell, 2006; Lazarus & Folkman, 1984) during difficult times. Furthermore, academics could be more likely to achieve WLB if they acquire resources such as time management skills, balance how they disburse their energy and learn to deal with emotional demands (Greenhaus & Powell, 2006; Rotondo & Kincaid, 2008). Pursuing these paths could help employees to achieve efficient WLB. Much also needs to be done by the organization to assist employees to achieve WLB. Previous studies (e.g., Amazue & Onyishi, 2016; Haar et al., 2019) suggest that when employees feel that their organization supports them, it enhances their WLB regardless of job type and family demands. This insight implies that Nigerian academics could better achieve WLB if they perceive their university to be supportive, for instance, the university management adjusting the calendar to lessen pressure on the part of these academics. This potential avenue underscores the importance of exposing supervisors to interventions programs that intended to promote their support toward employees (Kelly et al., 2014; Newman et al., 2015).

Our study also has implications for societal systems. The outbreak of the coronavirus forced employees to work from home, which entails combining work, home, and private life. As such, the coronavirus has profoundly influenced people’s “patterns of daily life” (Layton & Domegan, 2021, p. 4). As we highlighted in our research, one particularly important change to many people’s life was that they now worked from home, which can lead to conflicts (Bakker et al., 2008) and potentially destabilize a social system (Layton & Domegan, 2021). That is, a system may need to recalibrate how work demands, now accomplished from home, are to be managed by organizations, its leaders, as well as employees. In fact, the current study revealed an important challenge in this pursuit: Work engagement negatively impacted WLB through increased home demands, which may pose threats to the larger society. Thus, one important broader implication for organizations and its leaders is that flexibility in terms of *when* occupational tasks are to be accomplished could be granted to employees who juggle both work and home demands. Then, employees could flexibly deal with home demands and catch up at work later in a day, thereby facilitating both demands and sustaining WLB. Moreover, fortunately, governments intervened in various ways during the pandemic, including rendering financial support to businesses and providing palliatives to cushion the effects of lockdowns on societies and its members. For instance, providing and subsidizing childcare could go a long way in preventing work–life conflicts among employees dealing with both work and home demands. Altogether, these efforts could help “provisioning systems” to sustain in times of crisis, thereby bringing stability to socio-economic, managerial, and consumer decision-making (Layton & Domegan, 2021). Layton and Domegan (2021) expect similar dynamics between different levels within a system during future crises, for instance, induced by climate change (e.g., droughts, fires, or general temperature). Therefore, our practical implications may help not only to navigate the current crisis but also future ones.

### Limitations and Suggestions for Future Research

The outcomes of the present investigation should be appreciated in light of its shortcomings. First, our data were generated exclusively from self-report (single source), an approach that gives room for common method variance (CMV; Podsakoff et al., 2003). Social desirability may have played a role. Yet, we adhered to our promise of anonymity of responses, which may have minimized potential problems with this type of bias. Second, the cross-sectional nature of our data does not permit the establishment of causality among the study variables. For instance, the relationships found in our study could as well be bi-directional. Although the present study offers some interesting insights, future research should consider longitudinal designs to address the issue of causality. Third, our sample was rather of the same kind — academics whose jobs are primarily focused on teaching and research as drawn from universities within the Southeastern region of Nigeria. This could hinder the generalizability of our findings because academics from other regions of Nigeria or more generally other populations may operate in different social contexts that could have a different impact on their WLB. We advocate that future research should examine academics from diverse universities across Nigeria to enhance the degree to which inference can be drawn from their result. The current study can also be expanded in the future by investigating how the age of children, spousal engagement, and the individual view of “my marriage as a partnership” influences the relationships described herein. In addition, interaction effects between home demands and work engagement, between individual and job features on WLB, as well as the moderation of the indirect effect of work engagement on WLB by home crafting, should be studied in the future.

## Conclusions

Despite the shortcomings of the study, it represents one of the earliest attempts at creating deeper knowledge on the link between the “dark side” of work engagement and WLB in a different context, Nigeria, and how home demands represent a mechanism through which this relationship occurred. Consequently, our study adds significantly to the scarcity of literature in the study of work engagement, home demands, and WLB in a neglected context — Nigeria. As WLB is an important issue among employees, and as sustaining WLB seems to be a global challenge that cut across various occupational groups, it becomes pertinent for researchers to consistently examine factors that promote or impede WLB, especially in developing countries where similar studies are lacking. Moreover, our study offers new approach in the indirect relationship between work engagement and WLB through the “new construct” – home demands, especially during COVID-19. Therefore, our research provides opportunities for future studies in this growing field of WLB research, thereby advancing these research avenues. Doing so is relevant as employees continue to regularly work from home in times of crisis, and as other opposing home demands can interject and pose risks to employees’ realization of WLB.

## Appendix

Below are statements that represent the experiences you have at home. Please use the scales *never, rarely, sometimes, and always* to indicate how often you have this experience.

### Items used to assess home demands

**Table:**

	Items	Never	Rarely	Sometimes	Always
1	Do you find that you are busy at home?	1	2	3	4
2	Do you have to do many things in a hurry when you are at home?	1	2	3	4
3	Do you have to carry out a lot of tasks at home [household/caring tasks]?	1	2	3	4
4	How often do emotional issues arise at home?	1	2	3	4
5	How often does your housework confront you with things that touch you personally?	1	2	3	4
6	How often do you get frustrated about things concerning your home-life?	1	2	3	4
7	Do you find that you have to plan and organize a lot of things in relation to your home life?	1	2	3	4
8	Do you have to remember a lot of things with regard to your home life?	1	2	3	4
9	Do you have to do many things simultaneously at home?	1	2	3	4
10	Do you have to coordinate everything carefully at home?	1	2	3	4

### Items used to assess work engagement

The following statements are about how you feel at work. Please, read each statement carefully and decide if you ever feel this way about your job. If you have never had this feeling, check (√) (never) in the box after the statement. If you have had this feeling, indicate how often you feel it by ticking in the box that best describes how frequently you feel.

**Table:**

	Items	Never	Almost never	Rarely	Sometimes	Often	Very often	Always
1.	At my work, I feel bursting with energy.	0	1	2	3	4	5	6
2.	At my job, I feel strong and vigorous.	0	1	2	3	4	5	6
3.	When I get up in the morning, I feel like going to work.	0	1	2	3	4	5	6
4.	I am enthusiastic about my job.	0	1	2	3	4	5	6
5.	My job inspires me.	0	1	2	3	4	5	6
6.	I am proud on the work that I do.	0	1	2	3	4	5	6
7.	I feel happy when I am working intensely.	0	1	2	3	4	5	6
8.	I am immersed in my work.	0	1	2	3	4	5	6
9.	I get carried away when I’m working.	0	1	2	3	4	5	6

### Items used to assess work –life balance

When I reflect over my work and non-work activities (your regular activities outside of work such as family, friends, sports, study, etc.), over the past three months, I conclude that:

**Table:**

	Items	Strongly disagree	Disagree	Neutral	Agree	Strongly agree
1.	I currently have a good balance between the time I spend at work and the time I have available for non-work activities.	1	2	3	4	5
2.	I have difficulty balancing my work and non-work activities.	1	2	3	4	5
3.	I feel that the balance between my work demands and non-work activities is currently about right.	1	2	3	4	5
4.	Overall, I believe that my work and non-work life are balanced.	1	2	3	4	5
